# Effects of symbiotic and vitamin E supplementation on blood pressure, nitric oxide and inflammatory factors in non-alcoholic fatty liver disease

**DOI:** 10.17179/excli2016-846

**Published:** 2017-03-20

**Authors:** Golnaz Ekhlasi, Mitra Zarrati, Shahram Agah, Agha Fatemeh Hosseini, Sharieh Hosseini, Shahrzad Shidfar, Seyed Soroush Soltani Aarbshahi, Elham Razmpoosh, Farzad Shidfar

**Affiliations:** 1 Department of Nutrition, School of Public Health, Iran University of Medical Sciences, Tehran, Iran; 2 Colorectal Research Center; Iran University of Medical Sciences, Tehran, Iran; 3 Department of Math and Statistics, School of Health Management and Information Sciences, Iran University of Medical Sciences, Tehran, Iran; 4 Department of Applied Chemistry, Faculty of Pharmaceutical Chemistry, Pharmaceutical Sciences Branch, Islamic Azad University, Tehran, Iran (IAUPS); 5 Internist, Worcester Memorial Hospital, University of Massachusetts, Worcester, Massachusetts, U.S.A; 6 Nutrition and Food Security Research Center, Shahid Sadoughi University of Medical Sciences, Yazd, Iran; 7 Department of Nutrition, Faculty of Health, Shahid Sadoughi University of Medical Sciences, Yazd, Iran; 8 Iran National Science Foundation, Tehran, Iran

**Keywords:** symbiotic, alpha-tocopherol, blood pressure, nitric oxide, NAFLD

## Abstract

Non-alcoholic fatty liver disease (NAFLD) has been suggested to be well correlated with altered blood pressure. This study was conducted to determine the effects of symbiotic and vitamin E supplementation on blood pressure and inflammatory indices of patients with NAFLD. This randomized, double-blind, placebo-controlled trial was performed among 60 NAFLD patients aged 25 to 64 years old. Participants were randomly divided into four groups to receive a 400 IU alpha-tocopherol and 2 × 10^8^ CFU/g symbiotic supplement for 8 weeks. The anthropometric parameters, systolic blood pressure (SBP) and diastolic blood pressure (DBP), serum malondialdehyde (MDA), nitric oxide (NO) and tumor necrosis factor α (TNFα) were assessed at baseline and after 8 weeks of intervention. After 8 weeks of intervention, combined symbiotic and alpha-tocopherol, symbiotic and alpha-tocopherol alone administration, compared with the placebo, resulted in significant decreases in SBP (-17.07±2.1, -16.07±3.56, -1.73±2.25 and -1.55±3.01 mmHg, P=0.01), serum MDA (-1.19±0.5, -0.12±0.65, 0.14 ± 0.64 and 0.16±0.34 nmol/mL, P<0.001), serum TNFα (-15.62±13.93, -9.24±7.12, -11.44 ± 15.47 and 3.01±1.71 pg/ml, P<0.001) concentrations. A significant decrease in serum AST (-11.36±4.52, -7.43±8.58, -5.93±6.61 and 2.5±5.75 μmol/L, P <0.001), ALT (-12.79±3.65, -3.66±6.81, -6.54±7.66 and 4.16±3.43 μmol/L, P <0.001) and ALP (-26.8±11.1, -4.56±9.22, -14.48±12.22 and 5.19±2.64 μmol/L, P <0.001) was seen. Variations in DBP and serum NO concentration were not significant. Alpha-tocopherol and symbiotic supplementation among patients with NAFLD resulted in decreased SBP, serum MDA, TNFα levels and enzymes liver; however, they did not affect DBP and serum NO concentration.

## Introduction

NAFLD is one of the most common chronic liver diseases in the world today, occurring when fat is deposited (steatosis) in the liver (Wong et al., 2014[46]). The prevalence of NAFLD is reported to be 14-30 % of the general population in different parts of the world (Adams and Angulo, 2006[1]); this condition affects 21.5-31.5 % of adult's population in Iran (Jamali et al., 2008[19]). Various factors including obesity, insulin resistance, hypertriglyceridemia, hypertension, protein-calorie malnutrition, rapid weight loss, the use of medications such as glucocorticoids and metabolic factors are reported as contributors to the increased risk of NAFLD (Adams and Angulo, 2006[1]).

Not only insulin resistance and oxidative stress status are considered as roots of non-alcoholic fatty liver disease, but also intestinal flora is responsible for NAFLD. Small intestinal bacterial overgrowth (SIBO) is proposed to be responsible for the inflammation occurred through the production of cytokine, yet the exact mechanism is still unknown (Medina et al., 2004[26]). Some particular risk factors such as obesity, diabetes mellitus (insulin resistance) and dyslipidemia can be strongly connected with NAFLD. 

Recent literature supports the role of a gut-liver interaction in the pathogenesis of portal hypertension as a novel therapeutic focus; the prevailing hypothesis is that bacterial translocation stimulates the release of pro-inflammatory cytokines and the activation of the vasodilator nitric oxide results in a more pronounced deterioration of the baseline hyperdynamic circulatory state (Chin-Dusting et al., 1997[9]; Wiest et al., 1999[44]).

Subjects with NAFLD and elevated liver enzyme levels are at an increased risk of developing blood pressure and metabolic syndrome due to the oxidative stress status; in fact, decreased level of antioxidants in patients with NAFLD has effects on the measures of blood pressure. TNFα is also responsible for the pathogenesis of NAFLD via effecting on mitochondrial radical formation which might finally lead to cell death (Tilg and Diehl, 2000[39]; Peralta and Rosello-Catafau, 2004[28]); on the other hand, intestinal flora has also a significant role in production of cytokines (Solga and Diehl, 2003[36]).

Recently, it has been suggested that immoderate proliferation of pathogenic intestinal bacteria is inhibited by probiotics; it has been well attested by animal and human researches that probiotic bacteria could control blood pressure due to its antihypertensive effects (Reid et al., 2009[32]). Some investigators have evidenced that probiotic bacteria may control blood pressure (Myhre et al., 2011[27]; van Baarlen et al., 2011[42]; Ebel et al., 2014[11]). This mechanism of action of probiotics is multifactorial, related to their impact on intestinal microflora, cytokine production and maintenance of the epithelial barrier (Boirivant and Strober, 2007[6]).

Further, antioxidant agents have been proposed as a potentially effective treatment; vitamin E is a potent antioxidant compound that has been tested in pediatric NAFLD due to the absence of side effects. Conflicting results have been reported in clinical trials, among both children and adults (Lavine, 2000[23]; Harrison et al., 2003[17]; Vajro et al., 2004[40]).

Despite these findings, to the best of our knowledge, there are no reports regarding the favorable synergistic effects of the symbiotic and alpha-tocopherol supplementation on biomarkers of inflammation, oxidative stress and blood pressure in NAFLD patients. We hypothesized that the combined supplementation with symbiotic and alpha-tocopherol might help NAFLD patients to control their blood pressure; the current trial was to assess the effects of some species of probiotics along with alpha-tocopherol supplements on biomarkers of inflammation, oxidative stress and blood pressure in NAFLD patients.

## Materials

### Participants

This randomized double-blind clinical trial was performed in Tehran, Iran, during 2012 to 2013 among 60 NAFLD patients (48 men and 12 women) aged 25 to 64 years old, who were recruited from Hazrat-e-Rasoul Medical Complex (Colorectal Research Center) in Tehran, Iran. 

### Ethics statements

This study was conducted in accordance with the Declaration of Helsinki and informed consent was obtained from all participants. The research was approved by the ethics committee of IUMS and was recorded in the Iranian website for registration of clinical trials (www.irct.ir: IRCT201111082709N22).

### Study design

**Figure 1:**
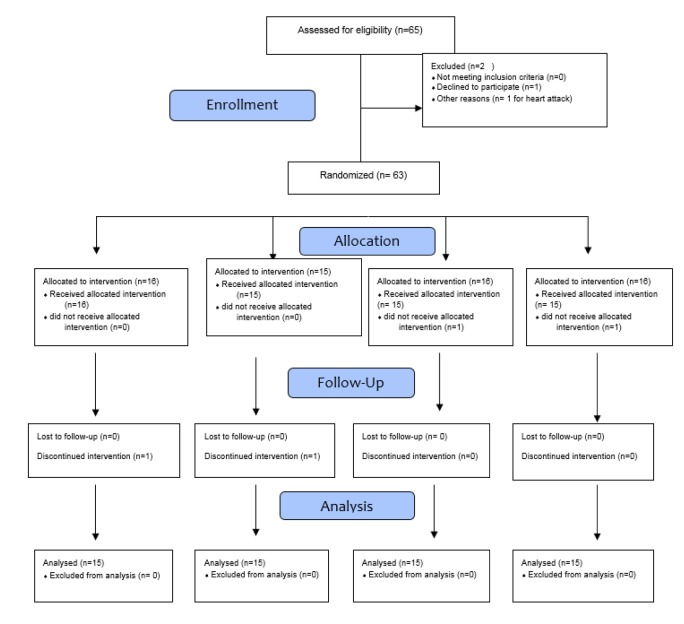
Flow diagram of patient recruitment and randomization process

All eligible patients with NAFLD were recruited (Figure 1(Fig. 1)). The diagnosis of NAFLD was based on hepatic ultrasonography (grade 1 to 3), which was associated with the elevation of alanine aminotransferase (ALT) concentration (30 mg/dL) for 6 months before the study initiation and at the time of randomization. The eligibility criteria included females and males with a body mass index (BMI) ranging from 25 to 35 kg/m^2^.

Exclusion criteria comprising any pathologic conditions affecting liver as viral hepatitis, alcohol consumption, hypothyroidism, Wilson disease, acute systemic disease, cystic fibrosis, coeliac disease and alpha-1-antitrypsin deficiency as well as a history of cancer, metabolic disorders, cardiovascular disease, autoimmune diseases and drug or alcohol abuse. Diabetes mellitus, pregnancy, lactation, menstruation at the time of blood sampling, infectious diseases during the study, using non-steroidal anti-inflammatory drugs, antibiotics, and probiotics and food supplements prior to the enrolment of the study were also considered as exclusion criteria.

Physical examinations, medical history, diet and physical activity level of each patient were assessed at the beginning of the study. Subjects were asked not to consume any probiotic containing food, yogurt, or its products during an initial 2 week run-in period before the study. They were also asked not to consume other probiotic products during the intervention. At the end of the run-in period, eligible patients were randomly assigned to one of the named groups. Symbiotic Group (n=15) who consumed symbiotic and alpha-tocopherol-like placebo capsule, alpha-tocopherol group (n=15) who received alpha-tocopherol and symbiotic-like placebo, symbiotic + alpha-tocopherol group (n=15) who consumed symbiotic and alpha-tocopherol supplementation and the final 15 patients were given symbiotic-like placebo and an alpha-tocopherol-like placebo supplementation as control group.

### Intervention

Each symbiotic capsule [Protexin; Probiotics International Ltd., Lopen Head, Somerset, United Kingdom] contained *Lactobacillus casei, Lactobacillus rhamnosus, Stretococcus thermophilus, Bifidobacterium breve, Lactobacillus acidophilus, Bifidobacterium longum, Lactobacillus bulgaricus* and prebiotic (fructooligosaccharide). The concentration of each probiotic strain was 2 × 10^8^ CFU/g per capsule. Patients were asked to consume 2 symbiotic capsules per day (each capsule contained 1 g) orally after the main meal. Two identical-appearing placebo capsules [corn starch, Zahravi Pharmaceutical Co, Tabriz, Iran] were taken daily by participants assigned to either the placebo or the alpha-tocopherol group. Justification for choosing this dosage was based on the earlier study (Aller et al., 2011[3]). Alpha-tocopherol [RRR-α-tocopherol, Zahravi Pharmaceutical Co, Tabriz, Iran] at a daily dosage of 400 IU and similar appearing placebo were administered orally. This chosen dose of alpha-tocopherol was similar to previous study in NAFLD patients (Chalasani et al., 2012[8]).

Randomization was carried out according to Balanced Block Randomization procedure while participants, nutrition specialists, and outcome assessors were all blinded to the interventions into which the individuals were allocated. This study was granted by Iran National Science Foundation.

### Treatment adherence

Compliance was monitored by phone calls weekly and verified using capsule counts (number of capsules left in the capsule bottle at the end of the study).

### Assessment of anthropometric measures

Weight (Seca, Hamburg, Germany) was measured at study baseline and after 8 weeks of intervention. Height was measured using a non-stretched tape measure (Seca, Hamburg, Germany). BMI was calculated using the height and weight measurements (weight in kg/[height in meters]^2^).

### Assessment of outcomes

The primary objective was a reduction in SBP and DBP in NAFLD patient with daily alpha-tocopherol and symbiotic supplementation; secondary objectives were changes in serum nitric oxide, MDA and serum TNFα. Levels of serum TNFα were measured using enzyme linked immunosorbent assay kits (eBioscience, USA). Plasma MDA levels were determined by thiobarbituric acid reactive substance spectrophotometric test (Janero, 1990[20]). Plasma nitrite/nitrate, taken as an index of nitric oxide (NO) concentrations, was determined using the Giess method which was modified by Tatsch et al. (2011[38]).

### Randomization

Randomization assignment was performed using computer-generated random numbers. Randomization and allocation were blinded to the researchers and participants until the final analyses were completed. The randomized allocation sequence as well as enrolling participants and allocating them to interventions were conducted by a trained staff at the department of nutrition.

### Statistical analysis

To examine the normal distribution of variables, we used Kolmogorov-Smirnov test. The analyses were conducted based on intention-to-treat (ITT) approach. One-way analysis of variance (ANOVA) was used to detect differences in general characteristics, dietary intakes, chemical biomarkers and blood pressure at the study initiation between the four groups. A Pearson χ2 test was used for comparison of categorical variables. Repeated measure ANOVA was used to determine the effects of symbiotic plus alpha-tocopherol administration on blood pressure and the used biomarkers, we adjusted the analysis for biochemical parameters, age and BMI, to assess their confounding effects. P values less than 0.05 were considered as statistically significant. All statistical analyses were done using the Statistical Package for Social Science version 17 (SPSS Inc., Chicago, Ill., USA).

## Results

The raw data are provided as an attachment (). 65 NAFLD patients were recruited in the study at baseline; however, 2 subjects were excluded as one of them was not a local resident (n = 1) and the other had a sudden heart attack during the study (n = 1); furthermore, in the symbiotic group, 2 subjects [death (1 = 3)], s (n = 1)] were excluded from the alpha-tocopherol group, due to personal reason and death. Moreover, 2 persons [death (n = 1) and withdrawal due to personal reasons (n = 1)] were excluded from the placebo group. Finally, 60 participants [symbiotic (n = 15), alpha-tocopherol (n = 15), symbiotic plus alpha-tocopherol (n = 15) and placebo (n = 15)] completed the trial. However, as the analysis was performed based on intention-to-treat principle, all 60 patients (15 in each group) were included in the final analysis. No side effects were recorded following the administration of symbiotic, alpha-tocopherol, and joint symbiotic and alpha-tocopherol supplements in patients with NAFLD throughout the study.

There were no initial differences in the mean measures of age (44 ± 20 y, P = 0.09), height, body weight, SBP, DBP and BMI between all the groups. Table 1(Tab. 1) summarizes the selected data of patients at the beginning and the end of the study. 

In addition, mean values of height, weight and BMI were not statistically different between the four groups after the treatment period.

According to the 3-day dietary records that were obtained throughout the intervention, no statistically significant differences were seen between the four groups in terms of macro- and micro-nutrient intakes (Table 2(Tab. 2)).

Joint supplementation with symbiotic and alpha-tocopherol had no significant effects on DBP and serum NO levels. After 8 weeks of intervention, all three groups of alpha-tocopherol only, individual symbiotic, and combined symbiotic and alpha-tocopherol experienced a significant improvements in SBP compared with the placebo group (P=0.01); however, improvements were much greater in the individual symbiotic (-16.07±3.56 mmHg) and combined symbiotic and alpha-tocopherol (-17.07±2.1 mmHg) groups compared with alpha-tocopherol group alone (-1.73±2.25 mmHg) (Table 1(Tab. 1)).

A significant decrease in serum MDA was shown in all intervention groups (alpha-tocopherol: 0.14±0.64; symbiotic: -0.12±0.65; combined group: -1.19±0.5 and placebo: 0.16±0.34 nmol/mL, P<0.001). Reduction in the levels of TNFα among individuals taking co-supplements of symbiotic and alpha-tocopherol, was greater in comparison to the other groups (alpha-tocopherol: -11.44± 15.47; symbiotic: -9.24±7.12; combined group: -15.62±13.93 and placebo: 3.01±1.71 pg/mL, P=0.001).

Furthermore, a significant decrease in serum AST level was seen among both individual and combined intervention with symbiotic and alpha-tocopherol groups (combined group: -11.36±4.52; alpha-tocopherol: -5.93± 6.61; symbiotic: -7.43±8.58; and placebo: 2.5±5.75μmol/L, respectively, P<0.001), ALT (combined group: -12.79±3.65; alpha-tocopherol: -3.66±6.81; symbiotic: -6.54± 7.66; and placebo: 4.16±3.43 μmol/L, respectively, P<0.001) and ALP (combined group: -26.8±11.1; alpha-tocopherol: -4.56±9.22; symbiotic: -14.48±12.22; and placebo: 5.19±2.64 μmol/L, respectively, P<0.001) (Table 3(Tab. 3)).

When the analyses were adjusted for baseline values including biochemical parameters, age and BMI, significant changes in our findings were observed except for NO (P = 0.82) and DBP (P = 0.82) levels (Table 4(Tab. 4)).

## Discussion

This study demonstrated that administration of symbiotic and alpha-tocopherol supplements for 8 weeks among patients with NAFLD had beneficial effects on SBP, liver enzymes, serum TNFα and MDA levels compared with the control group, while they did not affect DBP and serum NO levels. To the best of our knowledge, this is the first study evaluating the favorable synergistic effects of symbiotic and alpha-tocopherol supplementation on blood pressure and biomarkers of inflammation and oxidative stress among patients with NAFLD. 

Our study revealed that consumption of 2 × 10^8^ CFU/g symbiotic and 400 IU of alpha-tocopherol for 8 weeks among patients with NAFLD compared with the placebo group led to significant reductions in SBP.

In line with our findings, in a study by Yamamoto et al.(Yamamoto et al., 1994[47]) oral administration of *Lactobacillus acidophilus* yoghurt in spontaneously hypertensive rats (SHR) has shown that milk fermented with *L. heveticus *and casein hydrolysates, which were produced by this organisms, have anti-hypertensive activity. Cell wall polysaccharide-glycoprotein complexes of *L. casei* have also been reported to have antihypertensive activity in SHR and hypertensive rats (Sawada et al., 1990[35]; Furushiro et al., 1993[15]), it has been suggested that probiotic therapy may decrease blood pressure in cirrhosis via reducing intestinal permeability and bacterial translocation, decreasing the exposure of the immune system to intestinal bacteria and bacterial products and thereby reducing the inflammatory cytokines. Endotoxin is hypothesized to be originated from the intestinal tract as a result of increased gut permeability and bacterial translocation; in the study of Tandon et al. (2009[37]), there was a trend in the reduction of plasma endotoxin levels which was resulted by probiotic therapy, suggesting that probiotics would be effective in enhancing the gut barrier function, but the magnitude of change might have not been detectable by the intestinal permeability technique utilization.

Data on diastolic blood pressure needs to be interpreted with caution, and future studies with larger sample size would hopefully provide valuable information concerning this topic.

Findings from this study have shown that consumption of the symbiotic and alpha-tocopherol for 8 weeks among NAFLD patients significantly reduced serum TNFα compared with the placebo.

The beneficial effects of some probiotic species on serum TNFα concentration among patients with NAFLD have previously been evaluated (Aller et al., 2011[3]; Vajro et al., 2011[41]; Malaguarnera et al., 2012[25]). Supporting our study, a study by Malaguarnera et al. (2012[25]) reported significant decreases in serum TNFα levels after 24 weeks of intervention with *Bifidobacterium longum *and fructo-oligosaccharides among patients with non-alcoholic steatohepatitis. This protective effect is as a result of reduced hepatic exposure to intestinal products, such as lipopolysaccharides (LPS), which induced the release of TNFα from hepatic macrophage. TNFα is a cytokine that promotes cell death in the liver and increases inflammation and thus blood pressure (Solga and Diehl, 2003[36]).

It is logical to assume that similar effects of intestinal flora and NAFLD also exist, since TNFα is the key mediator of inflammation and so hypertension in this disease. Currently, there are two mechanisms in which intestinal flora may increase the hepatic oxidative stress and thus the blood pressure; the first is the increased endogenous ethanol production, and the second is the direct activation of inflammatory cytokines in luminal epithelial cells, non-parenchymal liver cells (macrophages) or both of them via releasing LPS, which may activate the production of TNFα in Kupffer cells and thus induce hepatic inflammation, oxidative stress and high blood pressure (Aqel and DiBaise, 2015[4]). 

High amounts of bacterial DNA and their derivatives induce the production of TNFα, IL-2, IL-6, IL-12 (Frances et al., 2004[13]; Frances et al., 2005[14]). In the liver, the extensive attachment of LPS to CD4/TLR4 induces high amounts of LPS-binding protein (Albillos et al., 2003[2]). Inefficient local immunity was demonstrated in liver disease, particularly in patients with cirrhosis; the potent mechanism is a depression in the activity of kupffer cells and the reticular endothelium system which plays a significant role in the defense against infected bacteria (Rimola et al., 1984[33]). Our findings differ from previous study due to TNFα levels; precisely, in the study by Tandon et al. (2009[37]) small increase observed in serum TNFα, was an unexpected finding after the probiotic therapy which could be anticipated to reduce the levels of pro-inflammatory mediators; although there were no significant adverse events of probiotic administration in patients with liver disease either in the current study or in previous ones (Rayes et al., 2002[30], 2005[31]; Lata et al., 2007[21]) there is a probability that an increase in the production of TNFα may represent harmful effects. There are two relevant subjects in this context, different types of probiotic species used in these studies and small sample size. 

In line with Loguercio et al. (2005[24]), the current study revealed that simultaneous supplementation of 400 IU alpha-tocopherol and symbiotic had synergistic effect on the reduction of serum MDA in NAFLD patients; the mentioned study demonstrated that supplementation of probiotic *VSL#3* in NAFLD patients could significantly improve plasma levels of MDA; another study has demonstrated that supplementation with *Lactobacillus casei *in the hyperlipidemic rats, decreased the levels of MDA (Zhang et al., 2010[48]).

Ejtehad et al. (2012[12]) showed that consumption of 300 g/d of probiotic yogurt containing *Lactobacillus acidophilus La5* and *Bifidobacterium lactis Bb12* could significantly decrease serum MDA concentration in type 2 diabetic patients; moreover, Daga et al. (2003[10]) found an inverse association between 400 IU/d vitamin E supplementation and serum MDA among COPD patients.

Oxidative stress is now believed to be an important factor in the development of hypertension in non-alcoholic fatty liver disease; increased blood pressure is found to be associated with NAFLD in patients with increased alanine transaminase concentrations (Lau et al., 2010[22]). Although, the mechanism explaining the association between NAFLD and increased blood pressure is still unclear, it has recently been demonstrated that there is a general thickening of the left ventricular wall and lipid peroxidation in NAFLD (Cassidy et al., 2015[7]); increased wall thickness due to the oxidative stress is associated with reduced longitudinal fiber shortening which is indicative for left ventricular hypertrophy (Hollingsworth et al., 2012[18]); this hypertrophy of the cardiac wall may also lead to the increased ventricular strain that is seen in NAFLD, which both finally affect the endocardium and entire wall as a result of the altered geometry (reduced radius).

This study assessed the supplementation of both symbiotic and alpha-tocopherol on serum NO concentration in people with NAFLD; although the plasma NO levels were increased in all intervention groups, the changes were not significant. The beneficial effects of vitamin E supplementation on serum NO and so blood pressure among patients with metabolic syndrome have been previously evaluated (Vivekananthan et al., 2003[43]; Rodrigo et al., 2008[34]).

Vasodilation in response to the increased flow is a function of the normal endothelium through the secretion of NO; loss of normal endothelial function results in impaired vasodilation and increased blood pressure and may be a common pathway to hypertension (Giles et al., 2012[16]). Antioxidants such as vitamin E have beneficial effects on controlling hypertension and reducing oxidative damage which result in a reduction in blood pressure levels (Baradaran et al., 2014[5]).

NO plays an important role in regulating systemic vascular resistance, arterial relaxation, and dispensability; collectively, these actions reduce cardiac hemodynamic load, which thereby reduce myocardial hypertrophy and left ventricular dysfunction and finally protect target organs (Raij, 2001[29]; Wilkinson et al., 2004[45]). Thus, NO plays a major role in maintaining and regulating blood pressure.

The strengths of the current study were the double blinding methods without any dropout rates. This study had some limitations, as well first of all, duration of intervention was relatively short. Moreover, we did not evaluate the effects of symbiotic and vitamin E supplementation on other factors related to blood pressure including rennin and angiotensin levels.

In addition, because of the budget limitations, we could not assess other biomarkers of systemic inflammation including interleukin 1(IL-1) and IL-6 as well as the biomarkers of oxidative stress such as catalase and superoxide dismutase in NAFLD patients. Future studies with crossover design, longer duration of intervention and larger sample size are needed to confirm the validity of our findings.

## Conclusion

Overall, administration of 400 IU alpha-tocopherol and symbiotic supplements containing *Lactobacillus casei, Lactobacillus rhamnosus, Stretococcus thermophilus, Bifidobacterium breve, Lactobacillus acidophilus, Bifidobacterium longum, *and* Lactobacillus bulgaricus* for 8 weeks among patients with NAFLD had beneficial effects on systolic SBP, MDA and TNFα but did not affect DBP and serum NO levels compared with the control group. Findings of the current study suggest that simultaneous supplementation of vitamin E and symbiotic may confer advantageous therapeutic outcomes for patients with NAFLD.

## Compliance with ethical standards

**Funding*: ***This study was funded by Iran national science foundation (grant number: 90005246).

**Conflict of interest: **The authors declare that they have no conflict of interest.

**Ethical approval:** All procedures performed in studies involving human participants were in accordance with the ethical standards of the institutional and/or national research committee and with the 1964 Helsinki declaration and its later amendments or comparable ethical standards.

**Informed consent:** Informed consent was obtained from all individual participants included in the study.

## Acknowledgements

The authors thank the participants in the study for their important contributions.

## Supplementary Material

Raw data

## Figures and Tables

**Table 1:**
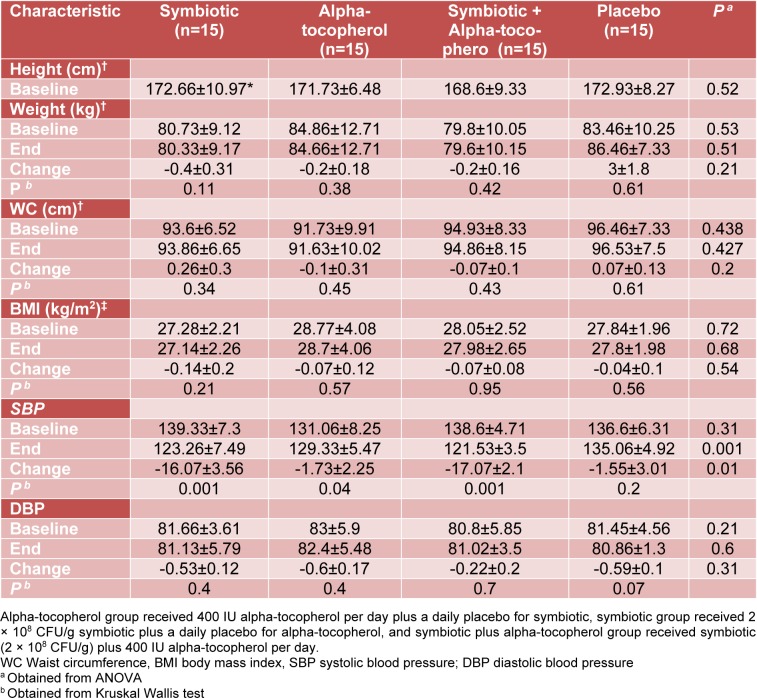
Baseline, end and change values of anthropometric indicators and blood pressure of study participants

**Table 2:**
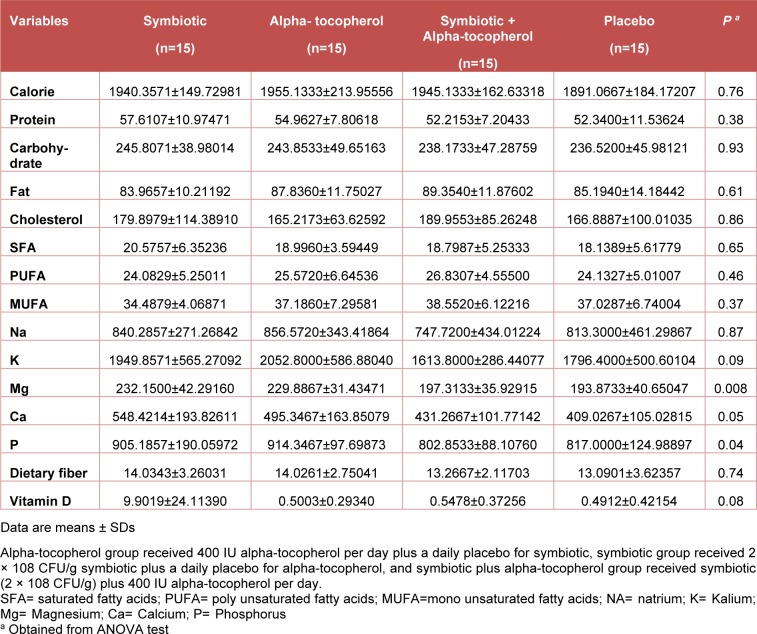
Dietary intakes of study participants throughout the study

**Table 3:**
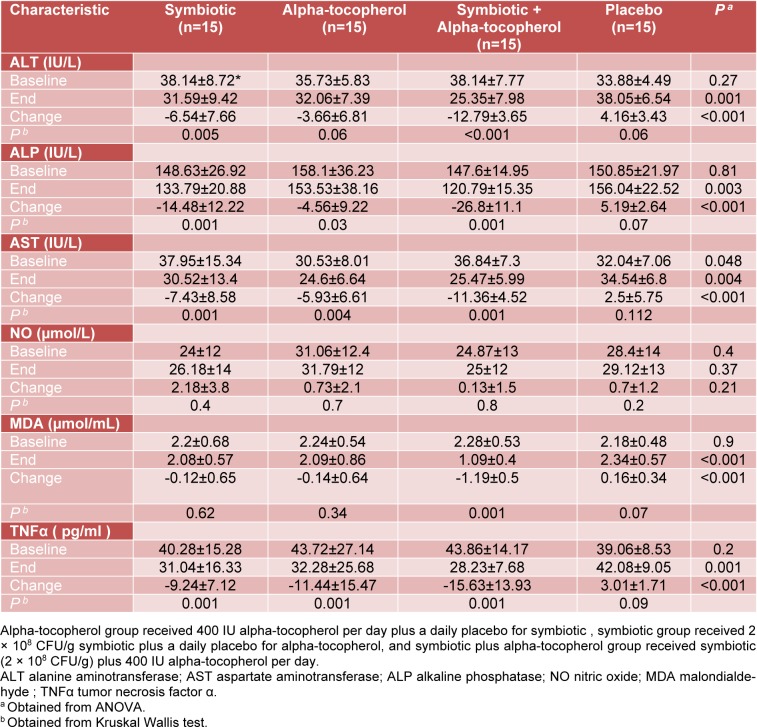
Baseline, end and change values of serum liver enzymes and some measured inflammatory factors of study participants

**Table 4:**
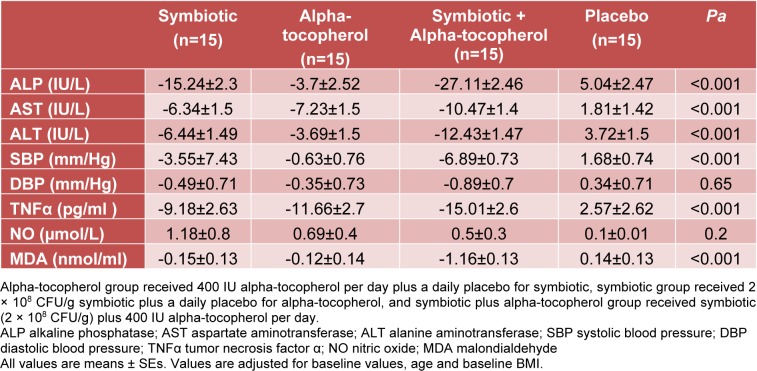
Adjusted changes in biochemical measurement and blood pressure in NAFLD patients that received symbiotic plus alpha-tocopherol, symbiotic and alpha-tocopherol supplements or placebo
