# Supplementary material for: Effects of symbiotic and vitamin E supplementation on blood pressure, nitric oxide and inflammatory factors in non-alcoholic fatty liver disease
Source: EXCLI J. 2017 Mar 20;16:278–90. doi: 10.17179/excli2016-846 (PMC5427479; doi:10.17179/excli2016-846)
Supplement: Raw data [file EXCLI-16-278-s-001.pdf]

raw data.sav

	sex	group	age	weight_B	weight_A	height
1	1.00	1.00	55.00	86.00	85.00	178.00
2	2.00	1.00	45.00	88.00	87.00	158.00
3	1.00	1.00	29.00	85.00	85.00	179.00
4	1.00	1.00	47.00	72.00	70.00	169.00
5	2.00	1.00	37.00	73.00	73.00	168.00
6	1.00	1.00	30.00	80.00	81.00	174.00
7	1.00	1.00	42.00	80.00	80.00	168.00
8	2.00	1.00	32.00	68.00	68.00	163.00
9	1.00	1.00	42.00	87.00	86.00	176.00
10	1.00	1.00	31.00	75.00	75.00	165.00
11	1.00	1.00	26.00	99.00	100.00	175.00
12	1.00	1.00	52.00	79.00	80.00	177.00
13	1.00	1.00	37.00	110.00	109.00	180.00
14	1.00	1.00	25.00	81.00	81.00	176.00
15	1.00	1.00	40.00	110.00	110.00	170.00
16	1.00	2.00	30.00	68.00	68.00	165.00
17	1.00	2.00	53.00	74.00	73.00	162.00
18	1.00	2.00	44.00	76.00	75.00	172.00
19	1.00	2.00	55.00	80.00	81.00	170.00
20	1.00	2.00	37.00	81.00	80.00	177.00
21	1.00	2.00	47.00	90.00	91.00	185.00
22	1.00	2.00	38.00	85.00	84.00	181.00
23	2.00	2.00	41.00	77.00	75.00	160.00
24	1.00	2.00	48.00	89.00	88.00	177.00
25	1.00	2.00	26.00	95.00	94.00	193.00
26	2.00	2.00	51.00	63.00	63.00	151.00
27	1.00	2.00	50.00	90.00	91.00	165.00
28	1.00	2.00	30.00	73.00	73.00	170.00
29	1.00	2.00	29.00	90.00	89.00	181.00
30	2.00	2.00	41.00	80.00	80.00	181.00
31	1.00	3.00	34.00	80.00	80.00	170.00
32	1.00	3.00	37.00	67.00	66.00	161.00
33	1.00	3.00	37.00	71.00	71.00	168.00
34	2.00	3.00	51.00	70.00	71.00	158.00
35	2.00	3.00	43.00	73.00	72.00	169.00
36	1.00	3.00	33.00	81.00	81.00	167.00

PDF created with pdfFactory Pro trial version www.pdffactory.com

raw data.sav

	BMI_B	BMI_A	waist_B	waist_A	ALP_B	ALP_A
1	27.14	26.83	90.00	90.00	200.00	203.00
2	35.25	34.85	93.00	93.00	203.00	201.00
3	26.53	26.53	83.00	83.00	193.00	185.00
4	25.21	24.51	90.00	90.00	171.00	163.00
5	25.86	25.86	81.00	81.00	204.00	196.00
6	26.42	26.75	85.00	86.00	184.00	175.00
7	28.34	28.34	92.00	92.00	125.00	115.00
8	25.59	25.59	82.00	81.00	116.50	112.50
9	28.09	27.76	93.00	92.00	117.00	109.00
10	27.55	27.55	90.00	90.00	104.00	102.50
11	32.33	32.65	111.00	111.00	161.00	150.00
12	25.22	25.54	83.00	82.50	122.00	110.50
13	33.95	33.64	112.00	112.00	168.00	150.50
14	26.15	26.15	87.00	86.50	179.00	200.00
15	38.06	38.06	104.00	104.50	124.00	130.00
16	24.98	24.98	85.00	85.00	167.00	161.50
17	28.20	27.82	99.00	99.00	120.00	112.50
18	25.69	25.35	98.00	98.00	189.50	171.50
19	27.68	28.03	97.00	97.00	119.00	110.50
20	25.85	25.54	88.00	88.00	160.00	145.50
21	26.30	26.59	89.00	89.50	112.00	119.50
22	25.95	25.64	90.00	90.00	169.00	152.60
23	30.08	29.30	92.00	91.50	191.00	142.00
24	28.41	28.09	105.00	105.00	124.00	109.00
25	25.50	25.24	97.00	97.00	150.00	140.50
26	27.63	27.63	95.00	99.00	117.00	110.50
27	33.06	33.43	105.00	105.00	129.50	109.50
28	25.26	25.26	84.00	84.00	175.50	151.50
29	27.47	27.17	91.00	91.00	156.50	140.10
30	24.46	24.46	89.00	89.00	149.50	130.20
31	27.68	27.68	90.00	90.00	153.50	135.50
32	25.85	25.46	85.00	85.00	134.00	113.00
33	25.16	25.16	90.00	90.00	165.00	102.00
34	28.04	28.44	92.00	92.00	178.00	157.50
35	25.56	25.21	88.00	88.50	134.50	114.50
36	29.04	29.04	89.00	89.00	156.50	130.00

PDF created with pdfFactory Pro trial version www.pdffactory.com

raw data.sav

	AST_B	AST_A	ALT_B	ALT_A	NO_B	NO_A
1	37.00	20.00	38.00	29.00	40.50	40.20
2	34.00	37.00	30.00	33.00	53.30	66.00
3	52.00	37.00	50.00	50.00	7.40	8.00
4	29.00	21.00	30.00	19.00	8.10	8.50
5	35.00	26.00	40.00	35.00	49.10	25.00
6	28.00	25.00	30.00	30.00	8.90	8.10
7	36.00	18.00	30.00	33.00	102.00	100.00
8	21.00	18.00	30.00	34.00	8.20	9.60
9	21.00	22.00	37.00	35.00	9.30	5.30
10	24.00	22.00	32.00	23.00	16.60	17.00
11	26.00	16.00	34.00	33.00	98.00	72.00
12	29.00	29.00	36.00	38.00	22.00	21.00
13	33.00	29.00	36.00	36.00	9.00	8.60
14	31.00	30.00	42.00	22.00	97.00	98.00
15	22.00	19.00	41.00	31.00	7.50	8.70
16	39.00	37.00	39.00	27.00	9.20	7.10
17	17.00	15.00	34.00	29.00	97.00	95.00
18	37.00	35.10	58.00	52.10	7.30	7.10
19	13.00	12.00	30.00	16.00	9.40	9.50
20	72.00	67.10	47.00	34.60	9.60	10.00
21	30.00	26.30	38.50	31.40	19.40	21.70
22	25.20	21.00	51.70	46.00	8.20	7.40
23	39.00	35.00	27.00	27.00	19.50	13.10
24	45.00	39.00	34.00	40.00	7.80	8.50
25	33.00	24.00	31.00	31.00	9.80	7.80
26	50.00	37.00	32.00	29.00	10.80	8.80
27	60.00	24.00	44.00	17.00	6.50	9.50
28	29.00	20.10	35.40	29.70	27.00	28.00
29	35.00	26.00	31.00	29.00	100.00	80.00
30	45.10	39.20	39.50	35.10	21.20	17.10
31	40.00	31.50	36.98	25.00	7.10	7.20
32	34.00	25.00	37.56	27.98	8.20	7.80
33	38.00	28.00	36.50	25.00	7.70	7.20
34	23.00	16.00	39.00	28.10	8.00	7.20
35	28.00	19.50	30.00	25.00	19.90	21.00
36	33.00	22.00	30.00	17.30	7.50	6.70

PDF created with pdfFactory Pro trial version www.pdffactory.com

raw data.sav

	MDA_B	SBP_A	MDA_A	TNF_B	TNF_A	SBP_B
1	2.30	131.00	2.00	36.30	30.10	135.00
2	1.90	131.00	1.70	24.90	19.60	130.00
3	2.00	120.00	1.60	35.10	11.30	125.00
4	2.20	125.00	2.20	23.40	17.00	122.00
5	2.70	133.00	2.20	20.00	15.10	140.00
6	2.20	136.00	2.60	20.10	18.20	140.00
7	1.30	137.00	1.40	77.80	24.60	140.00
8	1.20	133.00	.80	25.30	18.90	132.00
9	2.80	120.00	2.90	90.10	86.10	110.00
10	2.60	129.00	3.70	24.60	17.80	132.00
11	2.80	130.00	3.50	100.00	99.20	135.00
12	2.20	123.00	1.30	74.40	34.10	125.00
13	1.80	136.00	1.90	28.70	25.70	140.00
14	3.10	127.00	2.70	43.50	37.10	130.00
15	2.50	129.00	.90	31.70	29.50	130.00
16	3.34	125.00	2.78	37.50	25.50	128.00
17	1.67	133.00	2.12	33.50	27.10	135.00
18	1.30	132.00	1.92	29.40	26.20	130.00
19	1.50	125.00	1.87	35.10	10.40	128.00
20	3.00	127.00	3.20	29.00	29.60	130.00
21	2.98	110.00	1.50	29.60	24.87	115.00
22	1.80	126.00	2.12	44.30	37.43	130.00
23	3.00	130.00	2.12	37.20	20.00	134.00
24	1.89	120.00	.95	23.45	8.10	125.00
25	1.70	120.00	2.31	34.40	38.20	125.00
26	1.50	118.00	2.12	80.00	70.00	120.00
27	3.00	110.00	2.87	70.30	61.40	110.00
28	1.92	131.00	1.45	41.30	27.20	135.00
29	2.30	127.00	1.87	38.10	29.10	130.00
30	2.20	115.00	2.00	41.10	30.50	120.00
31	3.00	129.00	1.80	43.50	11.50	130.00
32	2.50	118.00	1.56	38.50	29.50	124.00
33	2.70	118.00	1.70	33.23	19.00	120.00
34	2.00	125.00	1.10	37.80	39.30	130.00
35	2.30	120.00	1.10	39.00	20.10	124.00
36	1.96	120.00	.89	92.70	34.20	135.00

PDF created with pdfFactory Pro trial version www.pdffactory.com

	DBP_B	DBP_A	diff_weight	diff_BMI	diff_waist
1	85.00	85.00	-1.00	-.32	.00
2	80.00	81.00	-1.00	-.40	.00
3	80.00	80.00	.00	.00	.00
4	80.00	80.00	-2.00	-.70	.00
5	90.00	89.00	.00	.00	.00
6	90.00	90.00	1.00	.33	1.00
7	90.00	90.00	.00	.00	.00
8	80.00	80.00	.00	.00	-1.00
9	70.00	70.00	-1.00	-.32	-1.00
10	80.00	81.00	.00	.00	.00
11	80.00	80.00	1.00	.33	.00
12	80.00	80.00	1.00	.32	-.50
13	90.00	80.00	-1.00	-.31	.00
14	80.00	80.00	.00	.00	-.50
15	90.00	90.00	.00	.00	.50
16	80.00	70.00	.00	.00	.00
17	80.00	85.00	-1.00	-.38	.00
18	80.00	80.00	-1.00	-.34	.00
19	80.00	72.00	1.00	.35	.00
20	90.00	90.00	-1.00	-.32	.00
21	85.00	90.00	1.00	.29	.50
22	80.00	80.00	-1.00	-.31	.00
23	80.00	80.00	-2.00	-.78	-.50
24	80.00	80.00	-1.00	-.32	.00
25	80.00	80.00	-1.00	-.27	.00
26	80.00	80.00	.00	.00	4.00
27	80.00	80.00	1.00	.37	.00
28	90.00	90.00	.00	.00	.00
29	80.00	80.00	-1.00	-.31	.00
30	80.00	80.00	.00	.00	.00
31	80.00	80.00	.00	.00	.00
32	70.00	70.00	-1.00	-.39	.00
33	80.00	80.00	.00	.00	.00
34	90.00	90.00	1.00	.40	.00
35	79.00	78.00	-1.00	-.35	.50
36	80.00	80.00	.00	.00	.00

PDF created with pdfFactory Pro trial version www.pdffactory.com

raw data.sav

	diff_ALP	diff_AST	diff_ALT	diff_NO	diff_SBP
1	3.00	-17.00	-9.00	-.30	-4.00
2	-2.00	3.00	3.00	12.70	1.00
3	-8.00	-15.00	.00	.60	-5.00
4	-8.00	-8.00	-11.00	.40	3.00
5	-8.00	-9.00	-5.00	-24.10	-7.00
6	-9.00	-3.00	.00	-.80	-4.00
7	-10.00	-18.00	3.00	-2.00	-3.00
8	-4.00	-3.00	4.00	1.40	1.00
9	-8.00	1.00	-2.00	-4.00	10.00
10	-1.50	-2.00	-9.00	.40	-3.00
11	-11.00	-10.00	-1.00	-26.00	-5.00
12	-11.50	.00	2.00	-1.00	-2.00
13	-17.50	-4.00	.00	-.40	-4.00
14	21.00	-1.00	-20.00	1.00	-3.00
15	6.00	-3.00	-10.00	1.20	-1.00
16	-5.50	-2.00	-12.00	-2.10	-3.00
17	-7.50	-2.00	-5.00	-2.00	-2.00
18	-18.00	-1.90	-5.90	-.20	2.00
19	-8.50	-1.00	-14.00	.10	-3.00
20	-14.50	-4.90	-12.40	.40	-3.00
21	7.50	-3.70	-7.10	2.30	-5.00
22	-16.40	-4.20	-5.70	-.80	-4.00
23	-49.00	-4.00	.00	-6.40	-4.00
24	-15.00	-6.00	6.00	.70	-5.00
25	-9.50	-9.00	.00	-2.00	-5.00
26	-6.50	-13.00	-3.00	-2.00	-2.00
27	-20.00	-36.00	-27.00	3.00	.00
28	-24.00	-8.90	-5.70	1.00	-4.00
29	-16.40	-9.00	-2.00	-20.00	-3.00
30	-19.30	-5.90	-4.40	-4.10	-5.00
31	-18.00	-8.50	-11.98	.10	-1.00
32	-21.00	-9.00	-9.58	-.40	-6.00
33	-63.00	-10.00	-11.50	-.50	-2.00
34	-20.50	-7.00	-10.90	-.80	-5.00
35	-20.00	-8.50	-5.00	1.10	-4.00
36	-26.50	-11.00	-12.70	-.80	-15.00

PDF created with pdfFactory Pro trial version www.pdffactory.com

	diff_DBP
1	.00
2	1.00
3	.00
4	.00
5	-1.00
6	.00
7	.00
8	.00
9	.00
10	1.00
11	.00
12	.00
13	-10.00
14	.00
15	.00
16	-10.00
17	5.00
18	.00
19	-8.00
20	.00
21	5.00
22	.00
23	.00
24	.00
25	.00
26	.00
27	.00
28	.00
29	.00
30	.00
31	.00
32	.00
33	.00
34	.00
35	-1.00
36	.00

PDF created with pdfFactory Pro trial version www.pdffactory.com

raw data.sav

	sex	group	age	weight_B	weight_A	height
37	1.00	3.00	39.00	72.00	71.00	165.00
38	1.00	3.00	42.00	83.00	83.00	174.00
39	1.00	3.00	42.00	102.00	103.00	180.00
40	1.00	3.00	57.00	70.00	71.00	158.00
41	1.00	3.00	51.00	90.00	89.00	185.00
42	2.00	3.00	64.00	85.00	86.00	158.00
43	1.00	3.00	30.00	95.00	94.00	186.00
44	2.00	3.00	36.00	81.00	79.00	160.00
45	1.00	3.00	27.00	77.00	77.00	170.00
46	1.00	4.00	36.00	73.00	72.00	170.00
47	1.00	4.00	45.00	78.00	79.00	173.00
48	1.00	4.00	46.00	85.00	86.00	171.00
49	1.00	4.00	52.00	85.00	85.00	181.00
50	1.00	4.00	39.00	84.00	83.00	179.00
51	1.00	4.00	37.00	80.00	80.00	169.00
52	1.00	4.00	30.00	88.00	86.00	178.00
53	1.00	4.00	60.00	78.00	77.00	172.00
54	2.00	4.00	57.00	67.00	67.00	160.00
55	1.00	4.00	31.00	110.00	109.00	187.00
56	1.00	4.00	60.00	87.00	88.00	177.00
57	1.00	4.00	30.00	83.00	84.00	170.00
58	1.00	4.00	48.00	98.00	99.00	182.00
59	2.00	4.00	40.00	76.00	75.00	155.00
60	1.00	4.00	36.00	80.00	80.00	170.00
61	.	.	.	.	.	.

PDF created with pdfFactory Pro trial version www.pdffactory.com

raw data.sav

	BMI_B	BMI_A	waist_B	waist_A	ALP_B	ALP_A
37	26.45	26.08	90.00	90.00	147.50	131.20
38	27.41	27.41	91.00	91.00	159.00	133.00
39	31.48	31.79	110.00	110.00	147.00	117.00
40	28.04	28.44	88.00	88.00	139.00	110.50
41	26.30	26.00	102.00	102.00	128.00	106.50
42	34.05	34.45	111.00	110.50	160.50	132.20
43	27.46	27.17	95.00	95.00	143.00	113.00
44	31.64	30.86	105.00	104.00	122.00	101.00
45	26.64	26.64	98.00	98.00	146.50	115.00
46	25.26	24.91	84.00	83.50	126.00	130.00
47	26.06	26.40	101.00	101.00	122.00	134.00
48	29.07	29.41	102.00	102.00	135.00	140.00
49	25.95	25.95	89.00	89.00	150.00	155.00
50	26.22	25.90	100.00	100.00	167.00	173.00
51	28.01	28.01	90.00	90.00	175.00	179.00
52	27.77	27.14	100.00	99.00	146.00	149.50
53	26.37	26.03	94.00	94.00	141.00	146.70
54	26.17	26.17	88.00	88.00	155.00	159.00
55	31.46	31.17	109.00	109.00	199.50	207.50
56	27.77	28.09	99.00	100.00	182.30	191.00
57	28.72	29.07	89.00	89.00	155.00	157.00
58	29.59	29.89	106.00	106.50	143.00	145.00
59	31.63	31.22	102.00	103.00	135.00	139.00
60	27.68	27.68	94.00	94.00	131.00	135.00
61	.	.	.	.	.	.

PDF created with pdfFactory Pro trial version www.pdffactory.com

raw data.sav

	AST_B	AST_A	ALT_B	ALT_A	NO_B	NO_A
37	40.30	31.10	38.10	28.10	45.60	48.00
38	42.00	31.89	56.00	41.50	7.00	7.20
39	38.80	25.10	53.50	41.20	94.00	93.10
40	36.00	25.00	29.00	12.00	14.00	16.00
41	38.50	15.00	30.00	18.50	9.70	8.70
42	29.00	20.00	38.10	20.00	6.90	7.90
43	55.00	35.00	39.50	28.10	8.00	9.10
44	39.00	29.00	38.10	21.10	61.70	50.50
45	38.00	28.00	39.90	21.50	19.40	11.00
46	33.00	29.00	30.00	31.00	100.00	97.00
47	32.00	38.90	38.00	44.30	14.00	19.00
48	26.00	34.00	28.00	31.00	92.00	90.20
49	23.00	32.30	30.00	32.10	27.00	28.30
50	36.00	31.45	38.23	39.10	14.00	14.80
51	22.00	23.00	25.10	26.20	10.50	8.40
52	33.89	41.00	35.78	45.20	15.00	15.90
53	28.00	43.00	39.00	50.00	22.00	17.00
54	41.30	40.00	37.65	42.65	7.50	6.20
55	48.00	49.00	35.00	44.00	13.80	12.10
56	38.00	32.00	32.00	35.20	14.50	12.10
57	35.00	36.10	38.00	39.20	76.00	42.00
58	27.65	28.10	31.56	33.67	10.50	7.20
59	31.00	32.40	39.00	40.20	6.00	9.80
60	25.78	27.90	31.00	37.00	13.20	11.80
61	.	.	.	.	.	.

PDF created with pdfFactory Pro trial version www.pdffactory.com

	MDA_B	SBP_A	MDA_A	TNF_B	TNF_A	SBP_B
37	3.21	120.00	1.10	39.50	25.10	134.00
38	2.45	121.00	1.21	44.00	33.40	134.00
39	1.89	125.00	.91	34.56	28.45	135.00
40	1.56	121.00	.87	39.10	29.10	127.00
41	1.70	128.00	.91	41.10	30.20	129.00
42	2.85	120.00	1.40	36.30	20.00	124.00
43	1.65	119.00	.81	44.10	36.10	124.00
44	1.80	119.00	.90	49.50	32.50	131.00
45	2.75	120.00	.20	45.10	35.10	128.00
46	1.98	126.00	1.96	39.20	41.50	123.00
47	3.30	131.00	3.40	36.00	39.20	130.00
48	2.67	122.00	2.98	31.50	33.54	118.00
49	1.56	129.00	1.67	40.80	43.10	127.00
50	1.76	130.00	1.82	43.00	51.00	128.00
51	2.40	116.00	2.51	49.00	53.00	112.00
52	1.89	123.00	1.95	39.00	44.00	119.00
53	2.12	131.00	2.23	33.00	35.50	130.00
54	1.87	136.00	3.20	30.00	33.00	135.00
55	2.00	128.00	1.87	52.00	55.00	122.00
56	1.76	127.00	1.87	45.00	48.00	123.00
57	3.00	129.00	3.10	33.00	36.10	130.00
58	1.98	129.00	1.85	29.00	30.50	125.00
59	2.32	134.00	2.41	29.50	30.50	135.00
60	2.12	130.00	2.32	55.98	57.30	127.00
61	.	.	.	.	.	.

PDF created with pdfFactory Pro trial version www.pdffactory.com

raw data.sav

	DBP_B	DBP_A	diff_weight	diff_BMI	diff_waist
37	90.00	80.00	-1.00	-.37	.00
38	80.00	80.00	.00	.00	.00
39	88.00	87.00	1.00	.31	.00
40	80.00	80.00	1.00	.40	.00
41	80.00	80.00	-1.00	-.29	.00
42	85.00	85.00	1.00	.40	-.50
43	70.00	70.00	-1.00	-.29	.00
44	80.00	80.00	-2.00	-.78	-1.00
45	80.00	80.00	.00	.00	.00
46	80.00	80.00	-1.00	-.35	-.50
47	80.00	81.00	1.00	.33	.00
48	80.00	81.00	1.00	.34	.00
49	80.00	81.00	.00	.00	.00
50	80.00	79.00	-1.00	-.31	.00
51	80.00	81.00	.00	.00	.00
52	80.00	80.00	-2.00	-.63	-1.00
53	80.00	80.00	-1.00	-.34	.00
54	80.00	80.00	.00	.00	.00
55	80.00	80.00	-1.00	-.29	.00
56	83.00	82.00	1.00	.32	1.00
57	80.00	81.00	1.00	.35	.00
58	80.00	80.00	1.00	.30	.50
59	82.00	84.00	-1.00	-.42	1.00
60	80.00	83.00	.00	.00	.00
61	.	.	.	.	.

PDF created with pdfFactory Pro trial version www.pdffactory.com

	diff_ALP	diff_AST	diff_ALT	diff_NO	diff_SBP
37	-16.30	-9.20	-10.00	2.40	-14.00
38	-26.00	-10.11	-14.50	.20	-13.00
39	-30.00	-13.70	-12.30	-.90	-10.00
40	-28.50	-11.00	-17.00	2.00	-6.00
41	-21.50	-23.50	-11.50	-1.00	-1.00
42	-28.30	-9.00	-18.10	1.00	-4.00
43	-30.00	-20.00	-11.40	1.10	-5.00
44	-21.00	-10.00	-17.00	-11.20	-12.00
45	-31.50	-10.00	-18.40	-8.40	-8.00
46	4.00	-4.00	1.00	-3.00	3.00
47	12.00	6.90	6.30	5.00	1.00
48	5.00	8.00	3.00	-1.80	4.00
49	5.00	9.30	2.10	1.30	2.00
50	6.00	-4.55	.87	.80	2.00
51	4.00	1.00	1.10	-2.10	4.00
52	3.50	7.11	9.42	.90	4.00
53	5.70	15.00	11.00	-5.00	1.00
54	4.00	-1.30	5.00	-1.30	1.00
55	8.00	1.00	9.00	-1.70	6.00
56	8.70	-6.00	3.20	-2.40	4.00
57	2.00	1.10	1.20	-34.00	-1.00
58	2.00	.45	2.11	-3.30	4.00
59	4.00	1.40	1.20	3.80	-1.00
60	4.00	2.12	6.00	-1.40	3.00
61	.	.	.	.	.

PDF created with pdfFactory Pro trial version www.pdffactory.com

	diff_DBP
37	-10.00
38	.00
39	-1.00
40	.00
41	.00
42	.00
43	.00
44	.00
45	.00
46	.00
47	1.00
48	1.00
49	1.00
50	-1.00
51	1.00
52	.00
53	.00
54	.00
55	.00
56	-1.00
57	1.00
58	.00
59	2.00
60	3.00
61	.

PDF created with pdfFactory Pro trial version www.pdffactory.com

Column 1: sex; 1=male, 2= female

Column 2: group; 1= Alpha- tocopherol, 2=symbiotic, 3= Symbiotic + Alpha-tocopherol, 4=placebo

Column 3: age

Column 4: weight_B; weight (kg) before intervention

Column 5: weight_A; weight (kg) after intervention

Column 6: height

Column 7: BMI_B; body mass index before intervention

Column 8: BMI_A; body mass index after intervention

Column 9: waist_A; waist circumference after intervention

Column 10: waist_B; waist circumference before intervention

Column 11: ALP_B; serum alkaline phosphatase concentration before intervention

Column 12: ALP_A; serum alkaline phosphatase concentration after intervention

Column 13: AST_B; serum aspartate aminotransferase before intervention

Column 14: AST_A; serum aspartate aminotransferase after intervention

Column 15: ALT_B; serum alanine aminotransferase before intervention

Column 16: ALT_A; serum alanine aminotransferase after intervention

Column 17: NO_B; serum nitric oxide concentration before intervention

Column 18: NO_A; serum nitric oxide concentration after intervention

Column 19: MDA_B; serum Malondialdehyde concentration before intervention

Column 20: SBP_A; systolic blood pressure after intervention

Column 21: MDA_A; serum Malondialdehyde concentration after intervention Column 22:

Column 22: TNF_B; serum tumor necrosis factor alpha concentration before intervention

Column 23: TNF_A; serum tumor necrosis factor alpha concentration after intervention

Column 24: SBP_B; systolic blood pressure before intervention

Column 25: DBP_B; diastolic blood pressure before intervention

Column 26: DBP_A; diastolic blood pressure after intervention

Column 27: diff_weight; change between mean of weight_B and mean of weight_A

Column 28: diff_BMI; change between mean of BMI_B and mean of BMI_A

Column 29: diff_waist; change between mean of waist _B and mean of waist _A

Column 30: diff_ALP; change between mean of ALP _B and mean of ALP _A

Column 31: diff_AST; change between mean of AST _B and mean of AST _A

Column 32: diff_ALT; change between mean of ALT _B and mean of ALT _A

Column 33: diff_NO; change between mean of NO _B and mean of NO _A

Column 34: diff_SBP; change between mean of SBP _B and mean of SBP _A

Column 35: diff_DBP; change between mean of DBP _B and mean of DBP _A
